# Supplementary material for: A Novel, Cell-Compatible Hyaluronidase Activity Assay Identifies Dextran Sulfates and Other Sulfated Polymeric Hydrocarbons as Potent Inhibitors for CEMIP
Source: Cells. 2025 Jan 11;14(2):101. doi: 10.3390/cells14020101 (PMC11764312; doi:10.3390/cells14020101)
Supplement: Supplementary file 1 [file cells-14-00101-s001.zip › cells-3372120-supplementary.pdf]

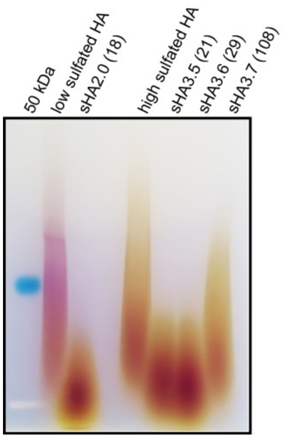

Supplementary Figure S1: Size estimation of sulfated HA. From different sulfated HA derivatives 10 µg were analysed by 2% agarose gel electrophoresis. Low- and high-sulfated HA from TCI chemicals was compared with sHA from Innovent with similar sulfation degree of 2.0 or 3.5 respectively.

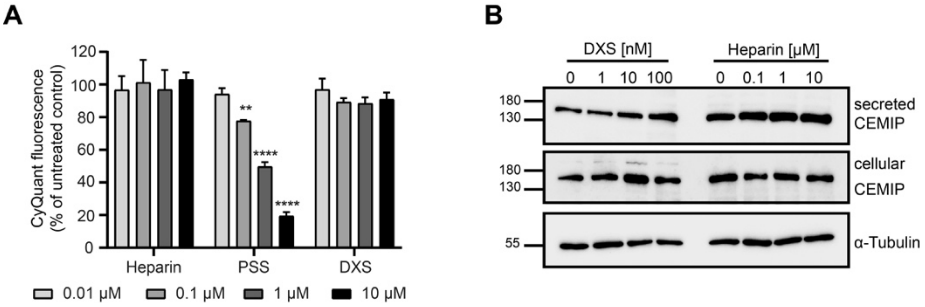

Supplementary Figure S2: Influence of heparin, PSS and DXS on cell numbers and CEMIP expression. 293T cells expressing human CEMIP were treated with the indicated concentrations of heparin, PSS or DXS 500 or were left untreated and were cultivated for 24 hours. **(A)** Cell numbers were assessed with CyQUANT Direct assay. Shown is the mean +/- SE of triplicate samples normalized to untreated controls (control = 100%). Significance was calculated using One-way ANOVA between treated and the untreated control group. **$p < 0.005$; ****$p < 0.0001$. **(B)** Cellular and secreted CEMIP levels were analysed in conditioned media or lysates of the cells by Western blot. Probing with α-Tubulin antibodies served as loading controls for the lysates.
